# *BRCA2*‐Related Hereditary Cancer Syndrome‐Associated Small Bowel Adenocarcinoma With Multiple *BRCA2* Mutations: A Case Report and Review of the Literature

**DOI:** 10.1002/cnr2.70200

**Published:** 2025-04-09

**Authors:** Francesca Antoci, Tommaso Colella, Elena Biletta, Erica Travaglino, Giuseppe De Lisi, Erica Quaquarini, Giovanni Arpa, Alberto Maria Pisacane, Myriam Katja Paris, Salvatore Corallo, Antonio Di Sabatino, Francesco Leone, Alessandro Vanoli

**Affiliations:** ^1^ Anatomic Pathology Unit IRCCS San Matteo Hospital Foundation Pavia Italy; ^2^ University of Pavia Pavia Italy; ^3^ Unit of Pathology, Department of Surgery ASL BI Nuovo Ospedale Degli Infermi Ponderano Italy; ^4^ Anatomic Pathology Unit, Department of Molecular Medicine University of Pavia Pavia Italy; ^5^ Medical Oncology Unit Fondazione IRCCS Policlinico San Matteo Pavia Italy; ^6^ Unit of Pathology Istituti Clinici Scientifici Maugeri IRCCS Pavia Italy; ^7^ Unit of Oncology, Department of Oncology Ospedale Degli Infermi Ponderano Italy; ^8^ First Department of Internal Medicine IRCCS San Matteo Hospital Foundation, University of Pavia Pavia Italy

**Keywords:** *BRCA2*, genetic tumor syndrome, small intestinal adenocarcinoma

## Abstract

**Background:**

Small bowel adenocarcinomas (SBAs) are rare and aggressive cancers. About one‐fifth of SBA patients have predisposing conditions; among them, there are also genetic tumor syndromes, including Lynch syndrome, familial adenomatous polyposis, and Peutz‐Jeghers syndrome. Although *BRCA2* mutations, both somatic and germline, have been recently described in SBAs, direct evidence of *BRCA2* inactivation in SBA tumor tissue of patients with *BRCA2*‐related hereditary cancer syndrome is still very limited.

**Case Presentation:**

Herein, we described a case of a 51‐year‐old woman with a history of breast cancer who developed an adenocarcinoma of the duodeno‐jejunal flexure causing persistent vomiting. After clinical staging, the patient underwent surgical resection, and histologic examination of the specimen confirmed a poorly differentiated adenocarcinoma infiltrating the visceral peritoneum and showing lymph node metastases (stage III, pT4N1). Two years later, the SBA relapsed, and next generation sequencing was performed in matched tumor and normal tissues. In addition to *KRAS* and *TP53* mutations in the tumor, both somatic and germline *BRCA2* mutations were identified, indicating biallelic *BRCA2* alterations.

**Conclusion:**

*BRCA2*‐associated hereditary tumor syndrome could have an etio‐pathogenetic role in SBA development; thus, we suggest that this syndrome should be considered in patients with an SBA diagnosis below the age of 50 years, especially when a personal or family history of breast cancer is present.

## 1 Introduction

Small bowel adenocarcinomas (SBAs) are rare cancers; however, their incidence is increasing, especially in the duodenal site. SBAs have a worse prognosis compared to colorectal cancers in all stages, and they are more often diagnosed at an advanced stage [1, 2, 3]. Risk factors for SBA include genetic tumor syndromes, immune‐related disorders, including Crohn disease and celiac disease, as well as lifestyle factors, such as alcohol abuse and smoking [4, 5]. Overall, predisposing conditions may be identified in a substantial proportion (about 20%) of SBA patients [6]. Interestingly, a recent multicenter international study showed that predisposing conditions were more frequently found in patients with early‐onset SBA (EO‐SBA), that is an SBA diagnosed in a patient younger than 50 years, compared to later‐onset SBAs [7]. In particular, EO‐SBAs were enriched in celiac disease patients.

Strong evidence suggests that several tumor genetic syndromes could be relevant in the pathogenesis of SBA [5]. Patients affected by Lynch syndrome have an SBA lifetime risk of 1%–4%, which is 25–291 times greater than that of the general population [8, 9]. Peutz–Jeghers syndrome (PJS) has also been related to SBA development, with a meta‐analysis showing that the relative risk of SBA in PJS compared to the general population is 520 [10]. Moreover, familial adenomatous polyposis (FAP) is a known risk factor for SBA, especially in the duodenum and ampulla [11]. In FAP patients, the risk of duodenal or ampullary cancer increases more than 100 times, reaching a prevalence of 1%–12% [12]. According to this evidence, the 2020 National Comprehensive Cancer Network (NCCN) recommends that all patients with small bowel cancer should be examined for their family history and considered for risk assessment [2].

A very recent study analyzing the prevalence of germline cancer susceptibility variants in patients with periampullary cancers showed that among 69 patients with duodenal cancer, 9 had a pathogenic germline variant, including three with a pathogenic/likely pathogenic germline variant in *BRCA2*. Additionally, germline mutations in *BRCA1*, *ATM, MLH1*, *MSH2*, *MSH6*, and *PALB2* were also identified, suggesting the importance of extending susceptibility gene testing to patients with non‐pancreatic periampullary carcinomas, including those with duodenal adenocarcinomas [13].

Here, we describe a unique case of SBA harboring multiple, somatic and germline, *BRCA2* mutations in a woman with an early‐onset adenocarcinoma of the duodeno‐jejunal flexure, suggesting a likely pathogenetic role of *BRCA2* biallelic mutations in the SBA development. Moreover, a literature review of *BRCA2* mutated cases was performed.

## 2 Case Description

We report the case of a 51‐year‐old woman with a history of uterine leiomyomas and early stage breast cancer, diagnosed in August 2018 (at the age of 45 years old) and treated with a total right mastectomy and axillary nodes dissection after instrumental staging that excluded the presence of distant metastases. Histological examination showed a no‐special‐type invasive breast carcinoma, grade 3, stage pT3N2 R0, according to WHO classification of breast cancer [14]. There was extensive lymphovascular invasion; up to 95% of the neoplastic cells exhibited expression of estrogen and progesterone receptors, whereas HER2 expression was negative (score 0). Ki67 proliferative index was 28%. As no family history suggestive of cancer predisposing syndromes was recorded, germline testing was not indicated according to current Italian guidelines. In October 2018, she started adjuvant chemotherapy (four cycles of doxorubicin and cyclophosphamide) followed by weekly paclitaxel for 12 administrations. In April 2019, endocrine therapy with exemestane was initiated in association with LHRH analogue due to the pre‐menopausal status.

**FIGURE 1:**
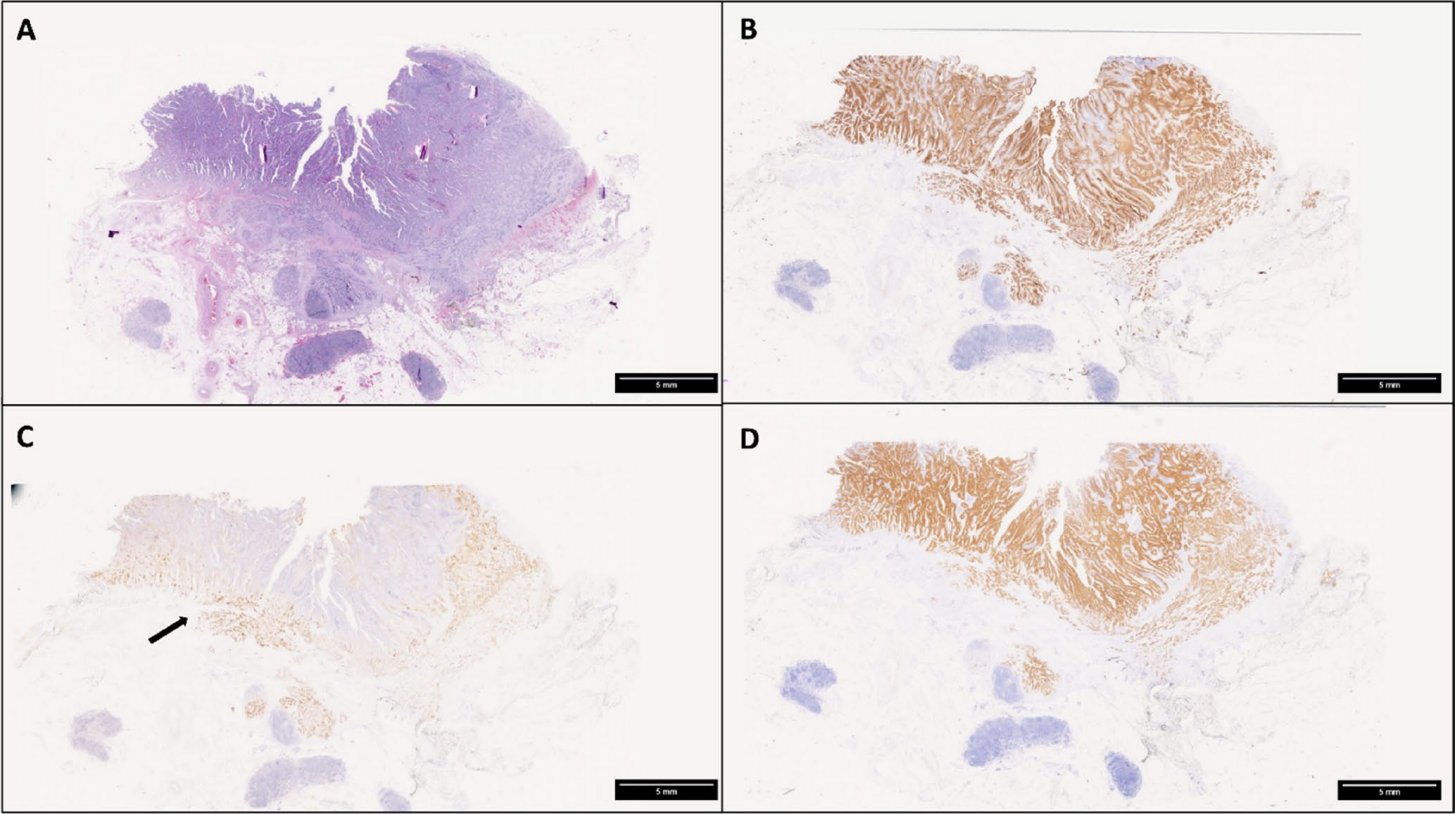
(A) Low power view of the small bowel adenocarcinoma (hematoxylin and eosin staining). (B–D) Tumor cells are diffusely positive for cytokeratin 20 (B, cytokeratin 20 immunostaining) and for CDX2 (D, CDX2 immunostaining), while the immunoreactivity for cytokeratin 7 is restricted to the deeper invasive portion (arrow) of the tumor (C, cytokeratin 7 immunostaining). Original magnification: 0.5× (A–D).

**FIGURE 2:**
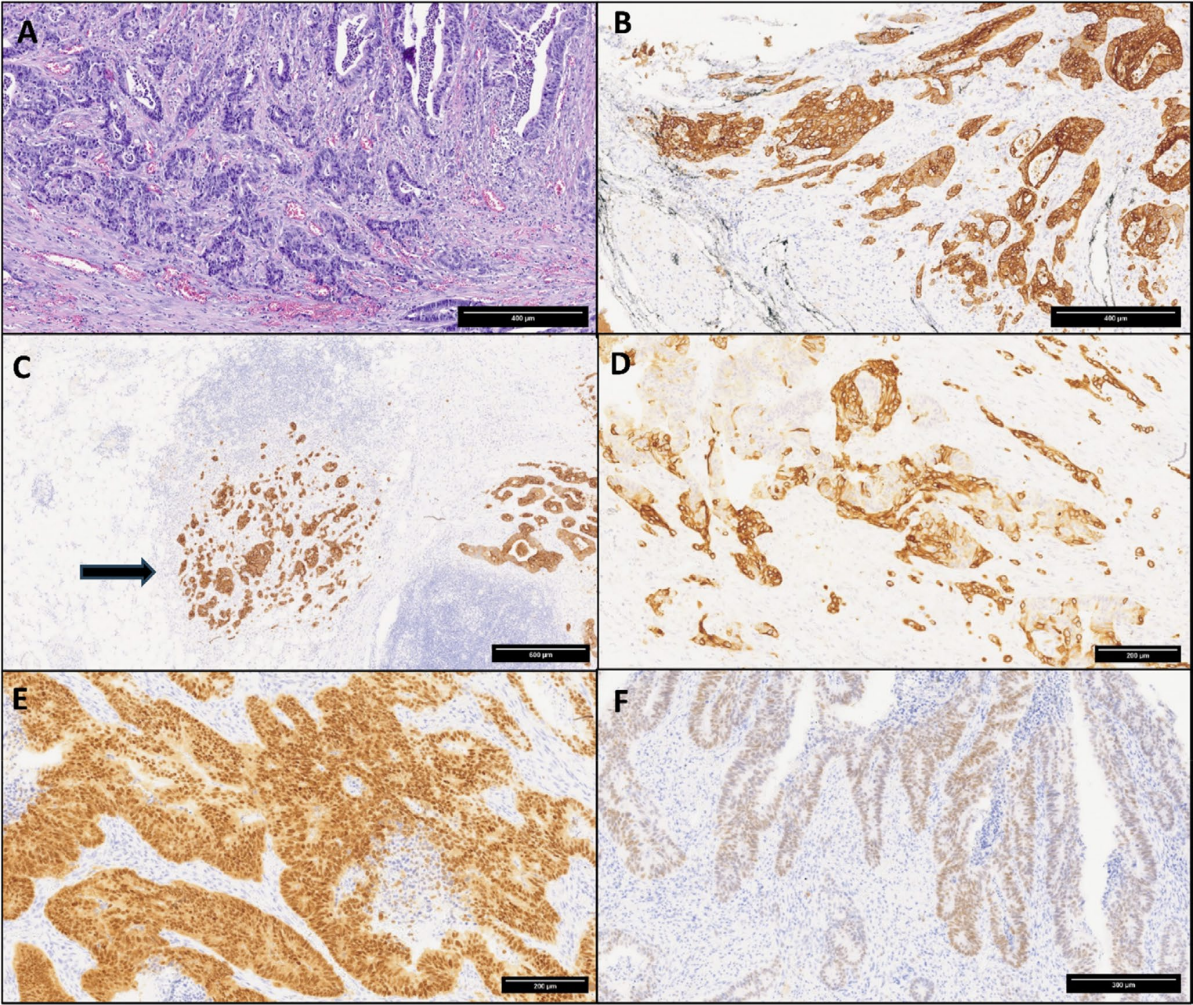
(A) High power view of the small bowel adenocarcinoma showing poor differentiation (hematoxylin and eosin). (B) Cytokeratin 20‐positive tumor cells perforating the serosa (cytokeratin 20 immunostaining). Note the black ink indicating the serosa surface. (C) Lymph node metastasis (arrow) of the cytokeratin 20‐positive small bowel adenocarcinoma (cytokeratin 20 immunostaining). (D) Tumor cells at the invasive front are also positive for cytokeratin 7 (cytokeratin 7 immunostaining). (E) The intestinal transcription factors CDX2 is diffusely positive (CDX2 immunostaining). (F) Several tumor cells are also weakly positive for the transcription factor SATB2 (SATB2 immunostaining). Original magnification: 10× (A); 10× (B); 5× (C); 15× (D); 15× (E); 13× (F).

In March 2020, at the age of 47 years, the patient was admitted to the Department of Oncology, Ospedale degli Infermi, Ponderano (BI), Italy, complaining of weight loss and persistent vomiting. Esophago‐gastro‐duodenoscopy was performed, showing an obstruction of the 4th part of the duodenum, and duodenal biopsies revealed an adenocarcinoma. Chest‐abdomen computed tomography (CT) confirmed a stricture of the duodeno‐jejunal flexure, without evidence of distant metastasis. In April 2020, the patient received a duodenal‐jejunal surgical resection, and histologic examination of the surgical specimen showed a poorly differentiated (G3) SBA with focal mucinous areas (Figures 1 and 2). The post‐operative pathological stage was pT4pN1, R0. Though perineural invasion was not detected, lymphovascular invasion and high‐score tumor budding (≥ 10 buds) were evident. Immunohistochemical stainings highlighted that neoplastic cells were diffusely positive for the intestinal markers cytokeratin (CK) 20 (90%) and CDX2 (80%), and focally positive for CK7; in addition, half of the cancer cells exhibited expression of the transcription factor SATB2 (Figures 1 and 2). PAX8, GATA3 and estrogen receptors were negative. Mismatch repair (MMR) proteins were tested by immunohistochemistry, resulting in an MMR‐proficient tumor. Tumor cells did not show membranous expression of HER2 (score 0 according to gastric cancer criteria [15]) and PD‐L1 immunohistochemical analysis revealed a tumor proportion score (TPS) < 1% and a combined positive score (CPS) < 1. From July 2020, adjuvant therapy with oxaliplatin and fluoropyrimidine (FOLFOX protocol for 6 months) was administered, but oxaliplatin was stopped in October 2020 due to neurotoxicity. Follow‐up CT scans were negative until April 2022, when a solid mass (50 × 40 mm) located at the right ovary was identified, with imaging features suggestive of a secondary tumor. Transvaginal ultrasound confirmed a right ovarian mass (36 × 26 × 27 mm) with no vascularization identified on color Doppler. Cancer markers tested were negative (CEA = 2.9 ng/mL, CA19.9 = 11.8 U/mL, and CA125 = 4.89 U/mL). In May 2022, a complete abdominal hysterectomy with bilateral salpingo‐oophorectomy was performed. Histological and immunohistochemical examination confirmed an ovarian metastasis from the previous SBA. Specifically, tumor cells expressed CK20, CDX2, CK7, and SATB2, while they were negative for PAX8 and WT1. A first line chemotherapy with capecitabine was then started in July 2022. In January 2023, a contrast CT revealed a peritoneal anterior paramedian contrast‐enhanced 13‐mm nodule, and an 18F‐fluorodeoxyglucose (FDG) PET (positron emission tomography)/CT scan performed in February 2023 showed multiple enhancing areas. After multidisciplinary team discussion, we assumed that clinical and radiological findings were diagnostic for peritoneal carcinomatosis, and peritoneal biopsy was not performed.

As part of an observational research project on EO‐SBA, in February 2023, targeted next‐generation sequencing (NGS) was performed to genetically characterize the primary SBA profile. We used an amplicon‐based multigene panel (AmoyDx HANDLE Classic NGS Panel) for the detection of different genomic alterations (single nucleotide variants (SNVs), insertions and deletions (InDels), gene fusions, copy number amplifications (CNAs) and microsatellite instability (MSI)) in 40 key solid tumor genes, using DNA and RNA isolated from formalin‐fixed paraffin embedded (FFPE) primary SBA specimens. Sequencing data were analyzed by AmoDx NGS data analysis system (ANDAS) to detect the genomic variants in the target region. No CNAs, gene fusions, or MSI status were detected. NGS analysis revealed two non‐silent and non‐intronic variants: (a) *KRAS*(NM_033360.4):c.35G>T;p.(G12V), a missense variation (variant allele frequency 28.25%) that occurs in exon 2 of the gene and is located in the P‐loop of the catalytic G‐domain of the protein; (b) *TP53*(NM_000546.5):c.772G>T;p.(E258*), a nonsense variation (variant allele frequency 60.91%) that occurs in exon 7 of the gene and leads to the production of several C‐terminally truncated protein forms.

Due to the personal cancer history of the patient and to her young age at SBA diagnosis, we decided to also evaluate *BRCA* genes, so we performed targeted NGS with an amplicon‐based multigene panel (AmoyDx HANDLE HRR NGS Panel). We used DNA isolated from FFPE tumor tissue specimens. NGS analysis revealed the following variants: (a) *BRCA2*(NM_000059.3):c.4119_4120delinsAT;p.(M1373_K1374delinsI*), an in‐frame substitution resulting in a stop codon (variant allele frequency 27.66%); (b) *BRCA2*(NM_000059.3):c.5645C>A;p.(S1882*), a nonsense variation (variant allele frequency 52.14%); (c) *BRCA2*(NM_000059.3):c.5918del;p.(N1973Ifs*31), a frameshift deletion variation (variant allele frequency 14.22%). All three variants occur in exon 11 of the gene.

In addition, small bowel non‐tumoral tissue was also tested to determine the somatic/germinal nature of the identified *BRCA2* variants. Only the *BRCA2*(NM_000059.3):c.5645C>A;p.(S1882*) nonsense variant was detected in both tissues (variant allele frequency: 48.93% in non‐tumoral tissue and 52.14% in tumor tissue), findings consistent with its germinal nature. Moreover, a confirmatory germline testing in a clinical laboratory (on peripheral blood cells) was offered and confirmed the *BRCA2* germline mutation. We also performed the NGS analysis on the breast cancer and identified the same *BRCA2*(NM_000059.3):c.5645C>A;p.(S1882*) nonsense variation as in the SBA and small bowel non‐tumoral tissue. Additionally, we identified the *PIK3CA*(NM_006218.4):c.3140A>G;p.(H1047R) missense mutation (variant allele frequency 74.55%) located in exon 21 of the gene in the breast cancer tissue.

In March 2023, FOLFIRI + bevacizumab treatment was started for 12 cycles. Subsequent instrumental evaluations showed partial radiologic response with a residual slightly enhancing area on the sigmoid area.

In November 2023, a peritoneal disease progression was evident at CT scans. In December 2023, a capecitabine/oxaliplatin + bevacizumab regimen was started with a complete response. On June 2024, the patient is alive, free of disease after 50 months after surgical resection of the primary SBA, and she is continuing with clinico‐instrumental follow‐up. The patient has maintained a good quality of life and remains independent in activities of daily living. The clinical course of the patient is summarized in Table 1.

**TABLE 1:** Timeline of the disease and treatment.

Timeline	Diagnosis	Treatment	Outcome
August 2018	No special‐type G3 breast invasive carcinoma (HR+; HER2; Ki67: 28%)	Right mastectomy (stage pT3pN2) + axillary nodes dissection + adjuvant chemotherapy + endocrine therapy	No evidence of disease recurrence
April 2020	Adenocarcinoma G3 of the duodeno‐jejunal flexure	Jejunal‐duodenal surgical resection (stage pT4pN1, R0) + adjuvant chemotherapy	Complete surgical resection; DFS: 24 months
April 2022	Ovarian metastasis from previous small bowel adenocarcinoma	Hysterectomy and bilateral adnexectomy + chemotherapy	Complete surgical resection; PFS: 9 months
January 2023	Peritoneal localization of small bowel adenocarcinoma	Chemotherapy + anti‐VEGF for 6 months)	Partial response; PFS: 3 months
November 2023	Peritoneal progression	Chemotherapy + anti‐VEGF (for 3 months)	Complete response, DFS: 4 months (last follow‐up in June 2024)

Abbreviations: DFS, disease free survival; HR, hormone receptor; PFS, progression free survival; VEGF, vascular endothelial growth factor.

## 3 Literature Review

We conducted a literature review to identify other *BRCA2*‐mutated SBA cases with a detailed description of their clinicopathologic features, and we found four cases. Their features are summarized in Table 2. In one case, the *BRCA2* mutation was somatic [16], while in the other three cases the *BRCA2* mutations were reported to be germline [13]. The median age at diagnosis for these patients was 60 years, which is lower than the median age of 66 years for SBA patients overall [8]. The duodenum appears to be the most affected small bowel site by *BRCA2*‐mutated SBAs. However, this site predilection needs to be confirmed, as there may be a selection bias. In one case, a concomitant *TP53* mutation was present. More importantly, in two out of four cases with *BRCA2* germline mutation, a personal and/or family history of breast cancer was recorded.

**TABLE 2:** *BRCA2* mutated non‐ampullary small bowel adenocarcinomas including a review of the literature and the present case.

References	Sex	Age	Site	Type of BRCA2 mutation	Co‐occuring gene mutations	MMR	Personal and/or family cancer history
Quaas et al. (2019)	NA	NA	Jejunum	Somatic (p.N986Ifs*5)	TP53 (p.R273C) KEAP1 (p.Y490C)	MSI	NA
Ando et al. (2024)	M	60	Duodenum	Germline (p.I2315Kfs*12)	NA	NA	Breast; uterus
Ando et al. (2024)	M	59	Duodenum	Germline (p.I1874Rfs*34)	NA	NA	None
Ando et al. (2024)	M	63	Duodenum	Germline (p.T2766Nfs*11)	NA	NA	Breast
Present case	F	47	Duodenal‐jejunal flexure	Germline (p.S1882*) and somatic p. (M1373_K1374delinsI*), p.(N1973Ifs*31)	KRAS (p.G12V), TP53 p.(E258*)	MSS	Breast

Abbreviations: MMR, mismatch repair; MSI, microsatellite instability; MSS, microsatellite stable; NA, not available.

## 4 Discussion

Due to the rarity of the tumor type, the molecular biology of SBAs is still poorly known. In a seminal study by Schrock et al., a distinct genomic profile of SBA emerged in comparison with colorectal and gastric cancers [17]. Although *TP53*, *KRAS*, *APC, SMAD4*, and *PIK3CA* are the most commonly mutated genes in both colorectal cancer and SBA, *TP53* and *APC* mutations are less frequent in SBA compared to large bowel carcinoma. In addition, MMR‐deficiency/MSI has been observed in a higher proportion of SBA cases in comparison with large bowel and gastric carcinomas [17].

In a Finnish cohort study investigating the somatic mutational landscape of microsatellite stable (MSS) SBAs, Hanninen et al. found novel candidate driver genes such as *BRCA2*, *ACVR1B*, *SMARCA4* [18]. Specifically, *BRCA2* somatic mutations were identified in 5% (5 out of 91) samples examined. A more recent genomic Chinese study confirmed a similar percentage (around 4%) for somatic [19] *BRCA2* mutations in SBA patients. Recently, Ando et al. [13] looked for germline susceptibility variants in patients with both pancreatic and non‐pancreatic periampullary cancer. Among the 395 non‐pancreatic periampullary cancers, they evaluated 69 duodenal cancers and found that 13% of them had a pathogenic germline variant. Specifically, three patients (4%) with duodenal carcinomas harbored a germline variant in *BRCA2* of clinical significance. Surprisingly, Casadei‐Gardini et al. [20], analyzed 24 Italian SBA patients and found *BRCA2* mutations in 29.4% of cases, without further details on the germline/somatic nature of the reported *BRCA2* mutations. In addition, they regrouped SBA patients in three different clusters. Specifically cluster 1 (29.2% of patients), defined as “immunological subtype”, exhibited significant correlations with MSI status, high tumor mutational burden (TMB), and celiac disease; cluster 2 (16.7% of patients), called “DNA Damage Repair (DDR)‐like”, presented with mutations in *BRCA2* in all cases and other genes involved in homologous DNA pairing and strand exchange, with potential benefit from poly (ADP‐ribose) polymerase (PARP) inhibitors (PARPi), and cluster 3 (54.1% of patients), called “Colon‐like”, characterized by genetic alterations in *TP53*, *KRAS*, *PIK3CA*, and *APC* [20].

*BRCA2* maps to chromosome 13q13.1 and, like *BRCA1*, encodes a tumor suppressor gene that regulates the homologous recombination repair of DNA double‐stranded breaks, preventing cell cycle arrest, apoptosis, and/or genomic instability [21]. Inactivating germline mutations in these two genes cause the *BRCA*‐related cancer predisposition syndrome, characterized by a significantly increased risk of breast cancer, tubo‐ovarian carcinoma, prostatic carcinoma, pancreatic carcinoma, primary peritoneal carcinoma, and also melanoma and other cancers at a low frequency [22, 23, 24]. The risk of SBA in patients with BRCA2‐related cancer predisposition syndrome is poorly known. Interestingly, *BRCA1/2* alterations were more frequently germline (57.4%) and biallelic (89.9%) in the “classic” *BRCA1/2*‐associated cancers (i.e., breast, ovarian, pancreatic and prostate cancers) compared to other cancer types (37.2% and 43.6%, respectively), including small intestine cancer [25]. *BRCA1/BRCA2* biallelic alterations lead to *BRCA1/BRCA2* loss of function, resulting in homologous recombination deficiency (HRD) which is targetable by PARPi and other DNA‐damaging agents.

To our knowledge, we described the first case of an SBA harboring three *BRCA2* variations, one of which is germline. In addition, we extrapolated from the literature review four patients with *BRCA2*‐mutated SBAs and well described genetic and clinico‐pathologic features [13, 16]: three harbored germline *BRCA2* mutations and the other one carried a somatic *BRCA2* mutation. Worthy of note, a personal or family history of breast cancer was present in three out of five patients with SBAs harboring *BRCA2* mutations (including our case). In two out of five cases reported in Table 2, a concomitant *TP53* mutation was present.

The tumoral immunophenotype (CK20+, CDX2+, CK7±, PAX8−, and GATA3−) of our case supported the intestinal origin of the ovarian metastasis. Interestingly, a significant fraction of tumor cells expressed the transcription factor SATB2. Although SATB2 is regarded as a lower gastrointestinal tract marker, it has also been reported to be expressed in about 20% of SBAs [26]. As we report in the case presentation, we can assert that the nonsense *BRCA2* alteration is germline because we also investigated the non‐tumoral tissue and found the same nonsense *BRCA2* variant (p.S1882*) as that found in the corresponding tumor. The other two variants, both somatic, were respectively an in‐frame substitution and a frameshift deletion. As reported by other authors [25], we categorized the alterations in *BRCA2* as biallelic because *BRCA2* mutations were multiple (three in our case). However, a limitation of our study is the absence of an assessment of the zygosity status of the *BRCA2* gene in the case described. This case confirms the susceptibility of *BRCA*‐deficient SBA to platinum‐based agents. Further investigations are needed to better clarify the predictive value of *BRCA2* mutations in SBAs, as well as the potential benefit of PARPi in patients with *BRCA‐mutated* SBAs. Moreover, a somatic screening for *BRCA2* variants at the time of cancer diagnosis could have influenced the medical management of our patients, including the consideration of risk‐reducing surgery and/or treatment with a PARPi.

## 5 Conclusion

We described a case of an EO‐SBA patient with both somatic and germline *BRCA2* variants, indicating a role of the *BRCA2*‐related hereditary cancer syndrome in the SBA development. This etiological association could have significant clinical implications both on screening and therapeutic approaches for patients and their relatives. Our case suggests that genetic counseling, along with *BRCA* germline testing and/or somatic screening for cancer susceptibility gene variants, should be offered to patients with EO‐SBA and to those with a personal or family history of breast cancer or other BRCA‐related cancers.

## Author Contributions

Conception and design: Francesca Antoci, Tommaso Colella, Elena Biletta, and Alessandro Vanoli. Data analysis and interpretation: Francesca Antoci, Tommaso Colella, Elena Biletta, Erica Travaglino, Giuseppe De Lisi, Erica Quaquarini, Giovanni Arpa, Alberto Maria Pisacane, Myriam Katja Paris, Salvatore Corallo, Antonio Di Sabatino, Francesco Leone, and Alessandro Vanoli. Manuscript preparation: Francesca Antoci, Tommaso Colella, and Erica Quaquarini. Manuscript revision: Elena Biletta, Erica Travaglino, Giuseppe De Lisi, Giovanni Arpa, Alberto Maria Pisacane, Myriam Katja Paris, Salvatore Corallo, Antonio Di Sabatino, Francesco Leone, and Alessandro Vanoli. Figure preparation: Giuseppe De Lisi, Erica Quaquarini, and Alessandro Vanoli. Approval of the final manuscript: Francesca Antoci, Tommaso Colella, Elena Biletta, Erica Travaglino, Giuseppe De Lisi, Erica Quaquarini, Giovanni Arpa, Alberto Maria Pisacane, Myriam Katja Paris, Salvatore Corallo, Antonio Di Sabatino, Francesco Leone, and Alessandro Vanoli.

## Consent

Only material that was not required for diagnosis was used and the patient signed a written informed consent for publication. All information regarding human material was managed using anonymous numerical codes, and all samples were handled in compliance with the Declaration of Helsinki.

## Conflicts of Interest

The authors declare no conflicts of interest.

## Data Availability

The data that support the findings of this study are available on request from the corresponding author. The data are not publicly available due to privacy or ethical restrictions.
